# Association between genetic polymorphism, severity, and treatment response among COVID-19 infected Egyptian patients

**DOI:** 10.3389/fphar.2023.1209286

**Published:** 2023-06-22

**Authors:** Abdelrahman Alaa, Neven Sarhan, Mohamed Gamal Lotfy El-Ansary, Naglaa Samir Bazan, Khaled Farouk, Raed Shahat Ismail, Mona Farag Schalaan, Adel R. A. Abd-Allah

**Affiliations:** ^1^ Clinical Pharmacy Department, Faculty of Pharmacy, Misr International University, Cairo, Egypt; ^2^ Critical Care Medicine Department, Kasr Al Ainy Hospital, Faculty of Medicine, Cairo University, Cairo, Egypt; ^3^ Pharmacology and Toxicology Department, Faculty of Pharmacy, Al-Azhar University, Cairo, Egypt; ^4^ Biochemistry Department, Faculty of pharmacy, Misr International University, Cairo, Egypt

**Keywords:** COVID-19, ACE2, ACE1, TMPRSS2, severity, genetic polymorphism

## Abstract

**Background:** The world has been suffering from the Coronavirus Disease-2019 (COVID-19) pandemic since the end of 2019. The COVID-19-infected patients differ in the severity of the infection and the treatment response. Several studies have been conducted to explore the factors that affect the severity of COVID-19 infection. One of these factors is the polymorphism of the angiotensin converting enzyme 2 (ACE-2) and the type 2 transmembrane serine protease (TMPRSS2) genes since these two proteins have a role in the entry of the virus into the cell. Also, the ACE-1 regulates the ACE-2 expression, so it is speculated to influence the COVID-19 severity.

**Objective:** This study investigates the relationship between the ACE-1, ACE-2, and TMPRSS2 genes single nucleotide polymorphism (SNPs) and the COVID-19 disease severity, treatment response, need for hospitalization, and ICU admission in Egyptian patients.

**Patients and Methods:** The current study is an observational prospective, cohort study, in which 109 total COVID-19 patients and 20 healthy volunteers were enrolled. Of those 109 patients, 51 patients were infected with the non-severe disease and were treated in an outpatient setting, and 58 suffered from severe disease and required hospitalization and were admitted to the ICU. All 109 COVID-19 patients received the treatment according to the Egyptian treatment protocol.

**Results:** Genotypes and allele frequencies among severe and non-severe patients were determined for ACE-1 rs4343, TMPRSS2 rs12329760, and ACE-2 rs908004. The GG genotype and the wild allele of the ACE-2 rs908004 and the mutant allele of the ACE-1 rs4343 were significantly more predominant in severe patients. In contrast, no significant association existed between the TMPRSS2 rs12329760 genotypes or alleles and the disease severity.

**Conclusion:** The results of this study show that the ACE-1 and ACE-2 SNPs can be used as severity predictors for COVID-19 infection since also they have an effect on length of hospitalization.

## 1 Introduction

Since the end of 2019, Egypt and the entire world have been suffering from the Coronavirus Disease 2019 (COVID-19) pandemic, which is caused by the severe acute respiratory syndrome coronavirus 2 (SARS-CoV-2). According to the World Health Organization (WHO), since the emergence of this new pandemic, there have been more than 767 million confirmed cases of COVID-19 patients; around 516 thousand of these cases are in Egypt (https://covid19.who.int/, accessed on 31 May 2023). The COVID-19 is a highly contagious infection that is transmitted mainly through respiratory droplets or direct contact, with the possibility of fecal–oral transmission (Jin et al., 2020). The incubation period of the infection ranges from 2 to 14 days with the symptoms appearing within an average of 4–7 days (Öztürk et al., 2020). The symptoms can vary from mild symptoms (low-grade fever, dry cough, fatigue, and myalgia) to severe and critical symptoms (respiratory distress and pneumonia) (Wang et al., 2020), which require hospitalization and sometimes admission to the Intensive Care Unit (ICU) and can lead to death. Several factors affect the severity of the symptoms experienced by COVID-19 patients. Studies concluded that gender (males), advanced age (over 65), ethnicity (African American race) and presence of co-morbid conditions like hypertension, dyslipidemia, cancer and others are associated with more severe symptoms (Adhikari et al., 2020; Wang et al., 2021).

One of the mechanisms of cell entry of the SARS-CoV-2 is that its spike protein (S) binds to the angiotensin-converting enzyme (ACE) 2 receptor, which is a transmembrane present in many epithelial cells in the body including the airway and the alveoli (Kamel et al., 2021; Gusev et al., 2022; Jackson et al., 2022; Rouaud and Méan, 2022). When the virus binds to the ACE-2 receptor, the type 2 transmembrane serine protease (TMPRSS2) enzyme activates the viral spike by cleaving it when it is attached to the ACE-2 receptor, making the fusion with the cell possible and promoting the virus to release its Ribonucleic acid (RNA) into the cell cytoplasm (Kamel et al., 2021; Gusev et al., 2022; Jackson et al., 2022). As the ACE-2 plays a vital role in the COVID-19 infection, the whole tissue-based renin-angiotensin system (RAS) is affected. The ACE-1 controls the formation of ACE-2, and the ACE-2 converts angiotensin 2 to angiotensin 1. During SARS-CoV-2 infection, the plasma ACE-1 activity is increased while ACE2 expression is decreased, which in turn causes the levels of angiotensin 2 to become unopposed (Zheng and Cao, 2020; Aladag et al., 2021; Yamamoto et al., 2021). This imbalance disrupts the hemostasis of the of body and may contribute to exacerbating the COVID-19 infection indirectly through inflammation and thrombosis (Aladag et al., 2021; Yamamoto et al., 2021), or causing a direct acute lung injury through different mechanisms like inducing the apoptosis of endothelial cells and of alveolar epithelial cells (Rarani et al., 2022) while boosting pro-inflammatory mediators like IL-6 and IL-8 (Suzuki et al., 2003). Along with its role in the viral cell entry, the TMPRSS2 activity also influences the Interferon-induced transmembrane protein 3 (IFITM3), which has an important role in the control of SARS-CoV-2 infection given its role in other viral diseases along with its interaction with the S protein (Shi et al., 2021; Dobrijevic et al., 2022). Given the involvement of the ACE-1, ACE-2 and TMPRSS2 in the COVID-19 infection, several studies have shown that there might be a strong correlation between the variability and genetic polymorphism of the ACE-1, ACE-2 and the TMPRSS2 genes and the risk and severity of the COVID-19 infection (Asselta et al., 2020; Hou et al., 2020; Jahanafrooz et al., 2022; Li et al., 2022).

The aim of the current study is to investigate if the variation in three different Single Nucleotide Polymorphisms (SNPs), one for the ACE-1 gene, one for the ACE-2 gene and another for the TMPRSS2 gene have any effect in the COVID-19 disease severity, treatment response and need for hospitalization and admission to the ICU in Egyptian patients.

## 2 Materials and methods

### 2.1 Study design and population

This is an observational, prospective cohort study which was conducted between December 2020 and June 2022. The total enrolled study subjects were 129 Egyptians, among which 58 were severe cases from Cairo University Hospitals, Cairo, Egypt, 51 non-severe cases who were treated in an outpatient setting and 20 healthy controls. Eligible patients for each group were those with established COVID-19 infection according to the WHO COVID-19 guidelines (Coronavirus Disease, 2019 COVID-19 Treatment Guidelines, 2021). Patients with severe disease were defined as Individuals who have SpO2 <94% on room air at sea level, a ratio of arterial partial pressure of oxygen to fraction of inspired oxygen (PaO2/FiO2) <300 mmHg, a respiratory rate >30 breaths/min, or lung infiltrates >50%. The study also included patients who had positive nasal swap for COVID-19 infection and their ages were between 18 and 80 years old. Patients with any prior respiratory problems such Asthma and chronic obstructive pulmonary disease (COPD) were excluded. The study was registered in ClinicalTrials.gov; Identifier: NCT05157217. The study protocol was approved by the Research Ethics Committee at the Faculty of medicine, Cairo University, Cairo, Egypt, and all participating patients provided written informed consent (REC number N-134-2020).

Patients who met the eligibility criteria were then divided into two groups; group 1 (Patients with Severe COVID-19 Disease): COVID-19 patients who suffered from serious respiratory complications that needed ICU admission and group 2 (Patients with non-severe COVID-19 Disease): COVID-19 carriers who were infected with SARS-CoV-2 and healed without suffering from any respiratory complications or the need for hospitalization. Patients were subjected to full laboratory including complete blood count (CBC), liver function and kidney function tests as well as inflammatory markers. Clinical and medical information were abstracted from the patient’s files and included age, sex, body weight, cigarette smoking status, medical history, and medications. All the patients received treatment according to the Egyptian COVID-19 treatment protocol (Table 1) established by the Egyptian Ministry of Health (Masoud et al., 2020). Whole blood samples (3 mL) were collected for DNA extraction and genotyping. The blood samples were stored at −80°C.

**TABLE 1:** Egyptian COVID-19 treatment guidelines.

Mild Disease			
Anti-Viral		Supportive Multivitamins	
Hydroxychloroquine 400 mg twice daily in the first day then 200 mg twice daily for 6 days **OR**.		Zinc 50 mg daily	
Ivermectin 36 mg on days 0-3-6 **OR**		Acetylcysteine 200 mg tid	
Favipiravir 1600 twice in the first day then 600 mg twice daily for 5-10 days		Lactoferrin one sachet bid	
		Vitamin C 1 gm dail	

Moderate Disease			
Anti-Viral	Anti-inflammatory	Anti-coagulations
Hydroxychloroquine 400 mg twice daily in the first day then 200 mg twice daily for 6 days.	Dexamethasone 6 mg or its oral equivalent (if respiratory rate >24 or CT scan shows rapid deterioration)	Parenteral Prophylactic: If D-Dimer 500-1000
Ivermectin 36 mg on days 0-3-6.	
Lopanivir /Ritonavir		Therapeutic Anticoagulation: If D-Dimer >1000
Remedesivir 200 mg in the first day then 100 mg daily for 5-10 days (for high-risk patients with SaO2≤94%)		

Severe Disease (Intermediate Care)			
Anti-Viral	Anti-inflammatory	Immunomodulators	Anti-coagulations
Remedesivir 200 mg in the first day then 100 mg daily for 5-10 days (for high-risk patients with SaO2≤94%)	Dexamethasone 6 mg	Convalescent Plasma (used before day 12)	Parenteral Prophylactic: If D-Dimer 500-1000
	Methylprednisolone 1 mg/kg/day		
Lopanivir /Ritonavir	Tocilizomab 4-8 mg/kg/day for 2 doses 12 to 24 hours apart (after failure of steroid therapy to improve the case for 24 hrs)		Therapeutic Anticoagulation: If D-Dimer >1000 or severe hypoxia

Critically Ill Patients (Intensive Care)			
Anti-Viral	Anti-inflammatory	Anti-coagulations
Remedesivir 200 mg in the first day then 100 mg daily for 5-10 days (for high-risk patients with SaO2≤94%)	Methylprednisolone 2 mg/kg/day or its equivalent	Therapeutic Anticoagulation
Lopanivir /Ritonavir	Tocilizomab 4-8 mg/kg/day for 2 doses 12 to 24 hours apart (after failure of steroid therapy to improve the case for 24 hrs)	

CT, computed tomography.

### 2.2 Genotyping procedure

DNA was extracted from peripheral blood leukocytes using the QIAamp DNA blood kits, cat no: 51,104 (Qiagen, Hilden, Germany). The procedure of extraction was by silica-membrane-based technology. Three genes’ variants were determined using TaqMan probe-based polymerase chain reaction (PCR). The Angiotensin‐converting enzyme gene (ACE) rs4343 (A2350G), Paired Ig-like receptors (PIR) gene G15494752T rs908004, and Transmembrane serine protease 2 Polymorphisms (TMPRSS2) gene rs12329760 C478G genotype variants were determined using Applied Biosystems™ ***TaqMan™ SNP Genotyping Assays*** “ACE rs4343 A/G, assay ID: C_11942562, PIR rs908004 G/T assay ID: C_8816953, and TMPRSS2 rs12329760 C/G, assay ID: C_25622353, purchased from (***ThermoFisher Scientific, Germany***). The PCR ***TaqMan Genotyping Master Mix*** kit, cat no: 4371353 (***ThermoFisher, Germany***). The appropriate volume from PCR reaction mix was prepared with a total volume of 10 µL/well. The reaction mix of each sample is composed of 5 µL of 2X TaqMan Genotyping Master Mix, 0.5 µL of TaqMan assay (20X), and 4.5 µL RNase free water. The thermal cycling protocol is optimized as follows” 950°C for 10 min for AmpliTaq Gold, UP Enzyme Activation, followed by denaturation step at 950°C for 15 s and annealing/extension at 600°C for 1 min for 40 cycles. The qPCR was performed on ***Applied BioSystems PCR instrument (ThermoFisher Scientific, Germany)***.

### 2.3 Data analysis

Continuous variables were represented as mean ± standard deviation. Categorical variables were presented as numbers and percentages. Hardy–Weinberg Equilibrium was tested using Chi-square test with one degree of freedom. Chi-square was used to compare the allele and genotype frequencies as appropriate. The association of genotypes with changes in endpoints was tested separately for each SNP. The three SNPs were tested in a dominant model. One-way ANOVA was used to analyze genotype group differences. We included covariates with a *p*-value <0.2 in a stepwise linear regression model. The binary logistic regression was used to investigate the association between the tested genotypes regarding outcomes of interest.

All reported *p* values were two-tailed, and a *p*-value of less than 0.05 was considered significant. Significant SNPs were determined according to the corrected Bonferroni *p*-value (*p* = 0.05/3 tested SNPs = 0.017, the Bonferroni-corrected significance threshold). All statistical analyses were carried out using Statistical Package for the Social Sciences (SPSS) software (version 22.0 for Windows; SPSS Inc., Chicago, Illinois, United States).

## 3 Results

**FIGURE 1:**
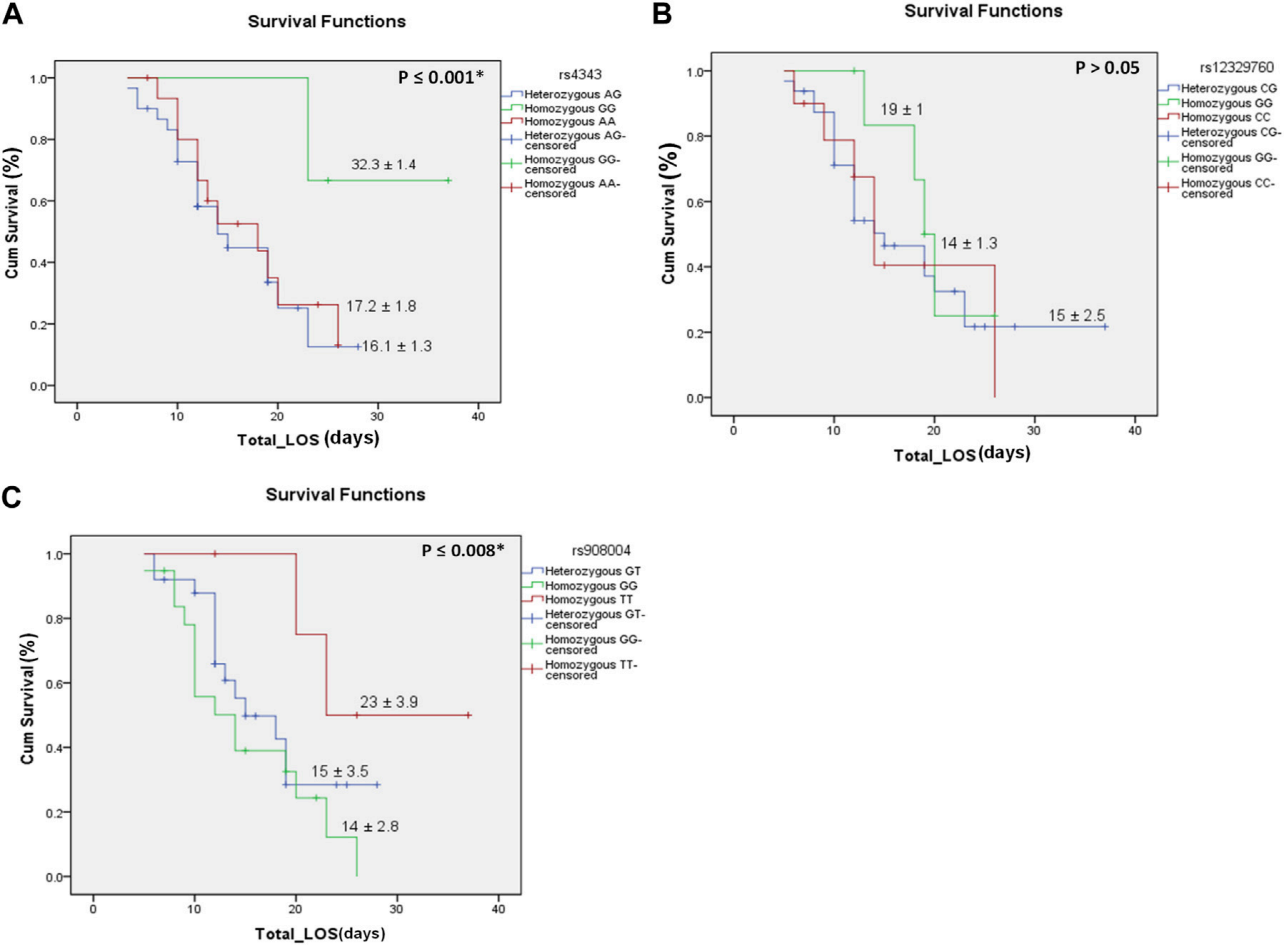
Kaplan-Meier survival *versus* the total length of stay in each of the three SNPs and their genotypes. **(A)** is for the ACE-1 rs4343genotypes, **(B)** is for the TMPRSS2 rs12329760 genotypes, **(C)** is for the ACE-2 rs908004 genotypes. Data is presented on the curve as median ± standard error. Significance levels at *p* < 0.05. The ACE-1 rs4343GG genotype was more significantly associated with longer hospitalization than the other genotypes. The ACE-2 rs908004TT genotype was more significantly associated with longer hospitalization than the other genotypes. **Total_LOS**, total length of stay presented as days; **Cum Survival,** cumulative survival presented as %.*Significance level at *p*-value <0.05.

The current study was conducted on 129 Egyptians who were sub-classed into three main groups: the COVID-19 severe patients (*n* = 58), the COVID-19 non-severe patients (*n* = 51) and healthy control (*n* = 20). The baseline characteristics for each group are presented in Table 2. There was no significant difference between the baseline characteristics of the severe, non-severe and healthy control groups except for the room air SO2, respiratory rate, C-reactive protein (CRP), procalcitonin and interleukin-6 (IL-6) levels. Genotyping was carried out in 3 SNPs for each patient in each group. The distribution of ACE-1 (rs4343), TMPRSS2 (rs12329760) and ACE-2 (rs908004) SNP genotypes among severe, non-severe COVID-19 patients and healthy control was analyzed. When comparing the distribution of the three SNPs’ genotypes among severe and non-severe patients, the ACE-2 rs908004 G/G genotype was found to be significantly more prevalent in severe patients (*n* = 21) than non-severe patients (*n* = 7), unlike the G/T genotype which was significantly more prevalent in non-severe patients (*n* = 39) than severe patients (*n* = 29). The ACE-1 rs4343 and TMPRSS2 rs12329760 genotypes, however, have shown no significant difference between severe and non-severe COVID-19 patients. These results are shown in Table 3. There was no significant difference between COVID-19 infected patients and healthy control regarding the three SNPs’ genotypes distribution, as presented in Table 4. When comparing the three beforementioned SNPs’ allele distribution among the two groups; the mutant allele of the ACE-1 rs4343 SNP and the wild allele of the ACE-2 rs908004 SNP were found to significantly more predominant in the severe COVID-19 patients than the non-severe COVID-19 patients, as shown in Table 5. The risk factors of ACE-2 rs908004 for COVID-19 severity calculated by linear logistic regression is shown in Table 6. In the severe group, the total and intensive care unit (ICU) length of stay (LOS) was compared to find if there is any association between the LOS and different genotypes of each SNP. The ICU LOS has shown no significant difference between the different genotypes of each SNP. When comparing the ACE-1 rs4343 SNP genotypes with the total LOS of severe COVID-19 patients, patients with the A/A genotype have significantly higher total LOS than the other two genotypes. Also, when comparing the ACE-2 rs908004 SNP genotypes with the total LOS in severe COVID-19, patients with the T/T genotype have significantly higher total LOS than the other two genotypes. The TMPRSS2 rs12329760 SNP genotypes seem to have no impact on the total LOS, since there is no significant difference in the total LOS between the three different genotypes. These results are shown in Table 7. There was no significant association between any of the SNPs and the patient’s mortality. A Kaplan-Meier survival function curve was plotted to further support these results and is illustrated in Figure 1. The combined genotypic effect of the ACE-1 rs4343 and ACE-2 rs908004 was compared with the different comorbidities that patients of both groups suffered from, and a linear-by-linear association was established and shown in Table 8. The combined genotypic effect was found to have a significant association with patients suffering from hypothyroidism. The other comorbidities show no significant association with the combined effect of the two SNPs.

**TABLE 2:** Baseline characteristics comparison between severe COVID-19 patients, non-severe COVID-19 patients, and healthy volunteers.

	Group 1 (severe patients)	Group 2 (non-severe patients)	Group 3 (healthy)	*p*-value
Patients characteristics				
Age (Mean ± SD)	62.36 ± 12.51	41.90 ± 13.49	54.9 ± 5.53	0.240
Male Gender (%)	53.6%	46.4%	50%	0.634
HTN (%)	52.4%	47.6%	42.5%	0.541
DM (%)	53.7%	46.3%	44.6%	0.577
CKD (%)	53.8%	46.2%	48.3%	0.612
ACS (%)	50%	50%	49.5%	0.589
Vital Signs				
HR (Mean ± SD)	94.48 ± 17.09	94.50 ± 3.04	85.44 ± 3.04	0.210
RBG (Mean ± SD)	251 ± 86.88	237.98 ± 78.52	240 ± 76.25	0.844
Room air SO_2_ (Mean ± SD)	76.32 ± 13.32	94.43 ± 13.38^a^	96.40 ± 10.50^a^	0.02*
RR (Mean ± SD)	35.57 ± 12.06	18.76 ± 8.17^a^	17.50 ± 9.18^a^	0.03*
Lab Profile				
Hg (Mean ± SD)	11.61 ± 2.02	11.61 ± 2.10	12.10 ± 2.16	0.829
TLC (Mean ± SD)	15.48 ± 29.95	17.13 ± 32.53	10.42 ± 30.56^ab^	0.035*
PLT (Mean ± SD)	2.45E2 ± 112.77	2.39E2 ± 119.96	2.58E2 ± 117.66	0.884
Urea (Mean ± SD)	91.44 ± 85.20	91.18 ± 92.86	91.18 ± 92.96	0.711
SCr (Mean ± SD)	2.42 ± 2.69	2.45 ± 2.80	2.35 ± 2.85	0.487
AST (Mean ± SD)	76.47 ± 105.97	72.96 ± 94.09	71.80 ± 95.10	0.142
ALT (Mean ± SD)	68.89 ± 179.75	75.72 ± 195.53	73.72 ± 198.32	0.761
Alb (Mean ± SD)	2.70 ± 0.58	2.70 ± 0.56	2.75 ± 0.59	0.267
INR (Mean ± SD)	1.31 ± 0.62	1.29 ± 0.55	1.30 ± 0.65	0.841
Ferritin (Mean ± SD)	2.09 ± 0.46	2.14 ± 0.66	2.15 ± 0.36	0.812
CRP (Mean ± SD)	1322 ± 106.57	138 ± 109.72^a^	7 ± 2.54^ab^	0.003*
D-Dimer (Mean ± SD)	3.67 ± 4.68	3.66 ± 4.86	0.54 ± 0.2^ab^	0.046*
Procalcitonin (Mean ± SD)	4.33 ± 15.46	1.85 ± 16.94^a^	0.46 ± 0.1^ab^	0.002*
IL6 (Mean ± SD)	2.55E2 ± 558.93	2.76E2 ± 92.85^a^	25.6 ± 10.50^ab^	0.004*

HTN, hypertension; DM, diabetes mellitus; CKD, chronic kidney disease; ACS, acute coronary syndrome; HR, heart rate; SO_2_, saturated oxygen; RR, respiratory rate; Hg, hemoglobin; TLC, total leucocytic count; PLT, platelet; SCr, serum creatinine; AST, aspartate aminotransferase; ALT, alanine transaminase; Alb, albumin; INR, international normalized ratio; CRP, C-reactive protein; IL6, interleukin-6.

*Significance level at *p*-value <0.05. ^a^Significant from Group 1. ^b^Significant from Group 2.

**TABLE 3:** Distribution of ACE-1 (rs4343), TMPRSS2 (rs12329760) and ACE-2 (rs908004) SNPs Genotypes among severe and non-severe COVID-19 patients. Statistical analysis done was chi-square test.

SNP/Variable	Genotypes						*p*-value; χ^2^
ACE- 1 (rs4343)	Homozygous GG		Heterozygous AG		Homozygous AA		
	No.	%	No.	%	No.	%	
Severe Patients (*n* = 58)	5	8.6	36	62.1	17	29.3	0.35; 2.16
Non-Severe Patients (*n* = 51)	9	17.7	30	58.8	12	23.5	

TMPRSS2 (rs12329760)	Homozygous GG		Heterozygous GC		Homozygous CC		
Severe Patients (*n* = 58)	9	15.5	36	62.1	13	22.4	0.91; 0.17
Non-Severe Patients (*n* = 51)	9	17.7	32	62.7	10	19.6	

ACE-2 (rs908004)	Homozygous GG		Heterozygous GT		Homozygous TT		
Severe Patients (*n* = 58)	21	36.2	29	50	8	13.8	0.01*; 5.39
Non-Severe Patients (*n* = 51)	7	13.7	39	76.5	5	9.8	

ACE, angiotensin converting enzyme; TMPRSS2, type 2 transmembrane serine protease.

*Significance level at *p*-value <0.05.

**TABLE 4:** Distribution of ACE-1 (rs4343), TMPRSS2 (rs12329760) and ACE-2 (rs908004) SNPs Genotypes among COVID-19 positive patients and healthy controls. The statistical analysis done was chi-square test.

SNP/Variable	Genotypes						*p*-value; χ^2^
ACE- 1 (rs4343)	Homozygous GG		Heterozygous AG		Homozygous AA		
	No.	%	No.	%	No.	%	
COVID-19 Patients (n = 109)	14	12.8	66	60.5	29	26.7	0.92; 0.16
Healthy Controls (*n* = 20)	13	65	2	10	5	25	

TMPRSS2 (rs12329760)	Homozygous GG		Heterozygous GC		Homozygous CC		
COVID-19 Patients (*n* = 109)	18	16.5	68	62.4	23	21.1	0.74; 0.59
Healthy Controls (*n* = 20)	11	55	4	20	5	25	

ACE-2 (rs908004)	Homozygous GG		Heterozygous GT		Homozygous TT		
COVID-19 Patients (*n* = 109)	28	25.6	68	62.3	13	22.1	0.88; 0.246
Healthy Controls (*n* = 20)	10	50	6	30	4	20	

ACE, angiotensin converting enzyme; TMPRSS2, type 2 transmembrane serine protease.

**TABLE 5:** Distribution of ACE-1 (rs4343), TMPRSS2 (rs12329760) and ACE-2 (rs908004) Alleles among severe and non-severe COVID-19 patients.

SNP/Variable	Alleles				*p*-value
ACE-1 (rs4343)	Wild allele		Mutant allele		
	No.	%	No.	%	
Severe Patients (*n* = 58)	23	39.7	35	60.3	0.005*
Non-Severe Patients (*n* = 51)	24	47.1	27	52.9	

TMPRSS2 (rs12329760)	Wild Allele		Mutant Allele		
Severe Patients (*n* = 58)	27	46.6	31	53.4	0.831
Non-Severe Patients (*n* = 51)	25	49	26	51	

ACE-2 (rs908004)	Wild Allele		Mutant Allele		
Severe Patients (*n* = 58)	35.5	61.2	22.5	38.8	0.006*
Non-Severe Patients (*n* = 51)	26.5	52	24.5	48	

ACE, angiotensin converting enzyme; TMPRSS2, type 2 transmembrane serine protease.

*Significance level at *p*-value <0.05.

**TABLE 6:** Risk factors for COVID-19 severity by binary logistic regression.

Risk factor	β^0^	*p*-value	Odds ratio	95%CI for Exp(B)
*ACE2 (rs908004)*	0.159	0.011	1.2	1.013–1.851

ACE, angiotensin converting enzyme.

**TABLE 7:** Comparison between total and ICU length of stay among the different genotypes in severe COVID-19 patients.

SNP	Genotypes			Significance *p*-value
ACE-1 (rs4343)	Homozygous GG	Heterozygous AG	Homozygous AA	
Total length of stay in days (Mean ± SD)	28.33 ± 7.57	13.57 ± 5.53	15.10 ± 6.71	0.001*
ICU length of stay in days (Mean ± SD)	17.67 ± 5.86	12.00 ± 4.89	12.59 ± 5.18	0.196

TMPRSS2 (rs12329760)	Homozygous GG	Heterozygous GC	Homozygous CC	
Total length of stay in days (Mean ± SD)	17.14 ± 4.67	14.97 ± 7.23	13.40 ± 5.89	0.358
ICU length of stay in days (Mean ± SD)	14.14 ± 4.85	12.41 ± 5.07	12.10 ± 6.06	0.694

ACE-2 (rs908004)	Homozygous GG	Heterozygous GT	Homozygous TT	
Total length of stay in days (Mean ± SD)	13.74 ± 6.10	14.44 ± 5.58	23.60 ± 9.13	0.008*
ICU length of stay in days (Mean ± SD)	11.84 ± 5.17	12.92 ± 5.02	13.80 ± 6.76	0.690

ACE, angiotensin converting enzyme; TMPRSS2, type 2 transmembrane serine protease; ICU, intensive care unit; SD, standard deviation. *Significance level at *p*-value <0.05.

**TABLE 8:** Association between ACE-1 and ACE-2 SNP combination and comorbidities.

SNP	Genotype	HTN %	DM %	NSTEMI %	CKD %	HT^a^ %	HT^b^ %	Hypopituitarism %	AKI %	RA %	OA %	Heart disease %	Liver disease %
Combined genotypic effect (ACE-1 rs4343 + ACE-2 rs908004)	0 (*n* = 40)	30	30	5	10	7.5	0	5	2.5	2.5	5	20	12.5
	1 (*n* = 30)	36.7	36.7	6.7	10	0	0	0	10	0	0	6.7	6.7
	2 (*n* = 26)	46.2	46.2	3.9	15.4	3.9	11.5	3.9	0	3.9	0	11.5	0
	3 (*n* = 8)	50	50	12.5	25	0	12.5	0	12.5	0	0	12.5	0
	4 (*n* = 5)	60	40	0	0	0	20	20	0	0	0	20	20
	Linear-by-Linear Association	3.255	1.740	0.001	0.261	1.375	8.409	0.378	0.008	0.038	2.095	0.326	1.095
	*p*-value	0.071	0.187	0.981	0.609	0.241	0.004*	0.539	0.930	0.845	0.148	0.568	0.295

ACE-1, rs4343 and ACE-2, rs908004 were combined as predictor for COVID-19, severity with patients’ comorbidities (coded as 0, 1, 2, 3, 4) (0 if rs4343 GG, and rs908004 GG, 1 if rs4343 GG, and rs908004 GT, or rs4343 AG, and rs908004 GG, 2 if rs4343 GG, and rs908004 TT, or rs4343 AG, and rs908004 GT, or rs4343 AA, and rs908004 GG, 3 if rs4343 AG, and rs908004 TT, or rs4343 AA, and rs908004 GT, 4 if rs4343 AA, and rs908004 TT). SNP, single nucleotide polymorphism; ACE, angiotensin converting enzyme; HTN, hypertension; DM, diabetes mellitus; NSTEMI, non-ST, elevation myocardial infarction; CKD, chronic kidney disease; HT^a^, hyperthyroidism; HT^b^, hypothyroidism; AKI, acute kidney injury; RA, rheumatoid arthritis; OA, osteoarthritis; *n*, number of patients. *Significance level at *p*-value <0.05.

## 4 Discussion

To this day, factors that affect COVID-19 clinical severity are still unraveled. Many studies attempted to investigate the relation between different gene polymorphisms and COVID-19 severity. The present prospective cohort study aims to assess the association between the ACE-1 rs4343, ACE-2 rs908004 and TMPRSS2 rs12329760 genes and the COVID-19 clinical severity in Egyptian patients. To our best knowledge, this is the first study to try to establish a relationship between these specific SNPs in these genes, the clinical severity of COVID-19 along with the total length of hospitalization and treatment response, especially in Egyptian patients. A total of109 patients were enrolled in the study, 58 patients suffered from severe COVID-19 disease and were admitted to the ICU, while 51 patients suffered from non-severe disease and were treated in an outpatient setting.

We found that there was a significant difference in the ACE-2 rs908004 genotypes and allele frequencies between the severe and the non-severe patients, where the GG genotype and the wild G-allele was more dominant in severe patients. We also found a significant association between the ACE-2 rs908004 polymorphism and the total length of hospitalization among clinically severe COVID-19 patients (Patients with GG genotype had significantly more total length of hospital stay than other genotypes). Several studies have reached a similar conclusion. In a study on German population, Möhlendick et al. (2021) found that the GG genotype or G-allele of ACE-2 rs2285666 was associated with patients that developed severe COVID-19 disease than the rest of the SARS-CoV-2 positive patient. In a recent Russian study, Shikov et al. (2020) found that several rare ACE-2 variants (for example, rs146598386, rs755766792 and others) influenced the clinical severity and outcome of COVID-19 disease. These results are also in agreement with Cafiero et al. (2021), which found that the ACE-2 rs2074192 SNP had a significant impact on disease severity. However, their results show that the T-allele was more dominant in symptomatic patients than asymptomatic patients, this difference from our results may be due to the difference in the measured SNP. In contrast to our findings, Karakaş Çelik et al. (2021) carried out a study on 155 COVID-19 patients in Turkey which showed that there is no correlation between the COVID-19 clinical severity and the ACE-2 rs2106809 and rs2285666 polymorphisms. Also, Gómez et al. (2020)’s results were not in agreement with our results, where they found that in 204 COVID-19 patients in Spain that there was no link between the ACE-2 rs2285666 SNP and the COVID-19 disease severity. These differences between our results and these two studies can be contributed to the difference in the studied SNP, ethnicity, or sample size.

The current findings revealed that there was no significant difference in neither the genotypes nor alleles of the TMPRSS2 rs12329760 between the severe and non-severe patients. This agreed with the findings German case-control study done by Schönfelder et al. (2021) on 239 COVID-19 positive patients. Their study results showed no correlation between neither TMPRSS2 rs2070788 nor rs12329760 polymorphism and the clinical severity of COVID-19. Also, Wulandari et al. (2021) found no association between the TMPRSS2 rs12329760 polymorphism and COVID-19 severity. In contrast to our findings, Monticelli et al. (2021) found a relation between TMPRSS2 rs12329760 polymorphism and COVID-19 severity and thus can help identifying individuals at risk of developing clinically severe COVID-19 disease in Italian patients. Also, in a study on Indian population, Ravikanth et al. (2021) found a significant link between TMPRSS2 rs12329760 variant and decreased COVID-19 disease severity.

Regarding the ACE-1 rs4343 assessed in the present study, there was no significant association between the SNP’s genotypes and COVID-19 disease severity. However, the mutant A-allele of rs4343 gene was significantly more dominant in non-severe COVID-19 patients than severe COVID-19 patients. We also found a significant association between the ACE-1 rs4343 polymorphism and the total length of hospitalization among clinically severe COVID-19 patients (Patients with TT genotype had significantly more total length of hospital stay than other genotypes). To our knowledge, all other studies conducted to test an association between ACE-1 gene and COVID-19 severity was on the ACE-1 insertion/deletion (I/D) polymorphism, however this is the first study attempting to investigate the association between an ACE-1 SNP and the COVID-19 severity. Two studies conducted by Möhlendick et al. (2021) and Karakaş Çelik et al. (2021) found no association between ACE I/D and COVID-19 disease severity. Another two studies by Cafiero et al. (2021) and Gunal et al. (2021) found that the ACE I/I allele was significantly more prevalent in asymptomatic COVID-19 patients, while the D/D allele was significantly more prevalent in symptomatic COVID-19 patients. Gómez et al. (2020) also found that depending on the hypertension status of COVID-19 patients, ACE I/D polymorphism can be associated with the risk of developing severe COVID-19 disease.

Other studies tried to find other predictive biomarkers for COVID-19 Severity. Ciaglia et al. (2021) found that long-lived individuals who are less susceptible to COVID-19 infection had higher levels of BPIFB4 protein circulating their blood than old healthy people and was even lower in COVID-19 infected patients. Sabbatino et al. (2021) suggests that the Programmed death-1 (PD-1) and its ligand programmed deathl-igand1 (PD-L1) has a potential prognostic role in COVID-19 and can be studied as target for future treatment.

In conclusion, our study demonstrated that the ACE-2 rs908004 genotype and allele as well as ACE-1 rs4343 allele can be a predictive factor for COVID-19 severity and treatment response in Egyptian patients. In contrast, ACE-1 rs4343 genotype and both TMPRSS2 rs12329760 genotypes and alleles were not associated to neither the COVID-19 severity nor treatment response in Egyptian patients. It could be concluded that the ACE-1 and ACE-2 variants were associated with an increased length of hospitalization in severe patients and thus require more aggressive approach and closer monitoring during treatment. We also found that there was no significant difference in the three SNPs’ genotypes between the COVID-19 infected patients and the healthy control.

Among the limitations of this study was the limited sample size and the study being single centered. Moreover, ACE-2 serum levels would have given us a better insight on predicting the severity and outcome of COVID-19. Unfortunately, no blood sample was left to perform this test. Further studies involving larger sample size and more diverse populations should be conducted to provide better understanding of the relationship between the ACE-1, ACE-2 and TMPRSS2 polymorphism and COVID-19 disease severity.

## Data Availability

The original contributions presented in the study are included in the article/supplementary materials,further inquiries can be directed to the corresponding authors.
